# Deciphering a pathogen’s evolution: a two-decade longitudinal study reveals virulence shifts and identifies durable *Pm* genes against Himalayan *Blumeria graminis* f. sp. *tritici* populations

**DOI:** 10.1007/s44154-026-00299-0

**Published:** 2026-05-09

**Authors:** Amritpal Mehta, Harneet Kaur, Daisy Basandrai, Ashwani Kumar Basandrai, Shafat Ahmad Ahanger, B. K. Sharma, Rajan Sharma, Sonali Sharma, Umer Basu

**Affiliations:** 1 Department of Plant Pathology, CSK Himachal Pradesh Agricultural University, Palampur, Himachal Pradesh 176062 India; 2 Department of Plant Pathology, Guru Kashi University, Talwandi Sabo, Punjab, 151302 India; 3 ICAR-National Institute of Biotic Stress Management, Baronda, Raipur, Chhattisgarh 493225 India; 4 State Key Laboratory of Crop Stress Resistance and High-Efficiency Production, Key Laboratory of Plant Protection Resources and Pest Integrated Management of Ministry of Education, Key Laboratory of Integrated Pest Management On Crops in Northwestern Loess Plateau of Ministry of Agriculture and Rural Affairs, College of Plant Protection, Northwest A&F University, Yangling, Shaanxi 712100 China; 5 CSK Himachal Pradesh Agricultural University, Research Sub Station, Una, Akrot, Himachal Pradesh 174303 India; 6 International Crop Research Institute for Semi-Arid Tropics (ICRISAT), Patencheru, Telangana 502324 India; 7 Division of Vegetable Science, Sher-E-Kashmir University of Agricultural Sciences and Technology of Jammu, Jammu and Kashmir, Chatha, 180009 India

**Keywords:** *Triticum aestivum*, Powdery mildew, *Blumeria graminis* f. sp. *tritici*, Resistance breeding, Pathotype, Virulence dynamics, Durability

## Abstract

Wheat powdery mildew, caused by the obligate biotrophic fungus *Blumeria graminis* f. sp. *tritici* (*Bgt*), is a devastating disease responsible for substantial global yield reductions. Host resistance offers the most sustainable management strategy, yet its durability is continually undermined by high evolutionary potential of the pathogen, driven by sexual recombination and rapid mutation. To decipher long-term virulence dynamics, we conducted a comparative analysis of *Bgt* populations in the Northwestern Himalayas over two decades (1994–1998 and 2015–2019). Pathotype profiling of 285 isolates on a common set of nine differential lines revealed a significant shift in virulence structure over time. While the mean virulence complexity per isolate increased in the contemporary population, indicating enhanced pathogenic capability, overall pathotype diversity decreased, suggesting a selective sweep toward fewer, more aggressive lineages. Marked changes in virulence frequency were observed with severe efficacy erosion for *Pm1a* (virulence increased from 6 to 63%) and *Pm8*, while virulence against *Pm2*, *Pm3a*, and *Pm3b* declined significantly. Notably, genes *Pm2* and *Pm4a* demonstrated complete and durable effectiveness throughout the study period. Our results identify these as stable resistance donors and underscores the continuous adaptation of *Bgt* populations. This study provides critical insights for designing durable resistance gene pyramids and underscores the necessity of continuous virulence surveillance to sustain wheat production in the face of evolving pathogen threats.

**Supplementary Information:**

The online version contains supplementary material available at 10.1007/s44154-026-00299-0.

## Introduction

Wheat powdery mildew, an economically devastating foliar disease of bread wheat (*Triticum aestivum* L.), is caused by the obligate biotrophic fungus *Blumeria graminis* f. sp. *tritici* (*Bgt*), and poses a significant and persistent threat to global wheat production. The severity of epidemics and the corresponding crop damage shows considerable variability, contingent upon regional climatic conditions. Empirical evidence from country-specific assessments highlights the magnitude of this threat, quantifying yield reductions up to 35% in Russia, 62% in Brazil, and 40% in China (Mehta 2014). A well-established correlation exists between disease pressure and productivity loss, with studies reporting reductions of 13–34% under moderate infection, escalating to 50–100% in severe outbreaks (Basandrai and Basandrai 2017; Basandrai et al. 2023). Further underscoring its pervasive impact, studies in the north-western Himalayas have recorded yield losses ranging from 8.7% to 41.3% (Rana et al. 2006).

The extent of yield and quality deterioration is critically dependent on the phenological stage at infection, with early-season onset causing the most significant reductions in both grain yield and quality parameters (Samobor et al. 2006). In this context, the cultivation of genetically resistant varieties is widely regarded as the most practical, economically viable, and environmentally sustainable management strategy. However, the long-term durability of this approach is consistently undermined by the evolutionary adaptability of *Bgt*. In temperate regions such as the dry temperate zone (Zone IV) of Himachal Pradesh, India, the pathogen completes its sexual cycle, forming chasmothecia that, upon maturation, release asci containing viable ascospores (Sharma et al. 1990). The process of sexual recombination genetically diversifies the pathogen population and facilitates the emergence of novel virulence combinations. Moreover, the capacity of pathogen for prolific asexual conidial production increases the probability of random mutations, enabling the rapid selection and dissemination of virulent lineages capable of overcoming host resistance within a few cropping cycles. Consequently, the deployment of cultivars with single-gene (vertical) resistance exerts strong selection pressure, which frequently accelerates the emergence of pre-dominant pathogenic races and induces rapid accumulation of virulent isolates (Akhtar et al. 2011; Wu et al. 2019; Mehta et al. 2024, 2025).

Continuous monitoring of pathogen population dynamics, including virulence profiling and tracking temporal shifts, is essential for forecasting resistance breakdown and sustaining the durability of resistant cultivars (Zeng et al. 2014; Wu et al. 2019). This necessity is important for wheat growing regions worldwide, including North Western Plain Zone (NWPZ) and North Hill Zone (NHZ) of India, due to the widespread cultivation of susceptible commercial varieties and the susceptibility observed in advanced breeding lines, which often lack high-level resistance (https://www.Aicrpwheatbarley.org/wp-content/uploads/2020/07/Crop-Protection-Report-2019-20_compressed.pdf). The current absence of a systematic, focused breeding program for *Bgt* resistance in India creates a critical imperative to identify and characterize effective resistance donors against prevailing virulence patterns. To address this gap, this study was designed to characterize the virulence patterns of *Bgt* populations across diverse agro-climatic zones of the Northwestern Himalayas and to analyze virulence dynamics over time, thereby providing valuable insights for developing durable resistant wheat varieties.

## Results

### Virulence structure of *Bgt* populations (1994–1998)

**Fig. 1:**
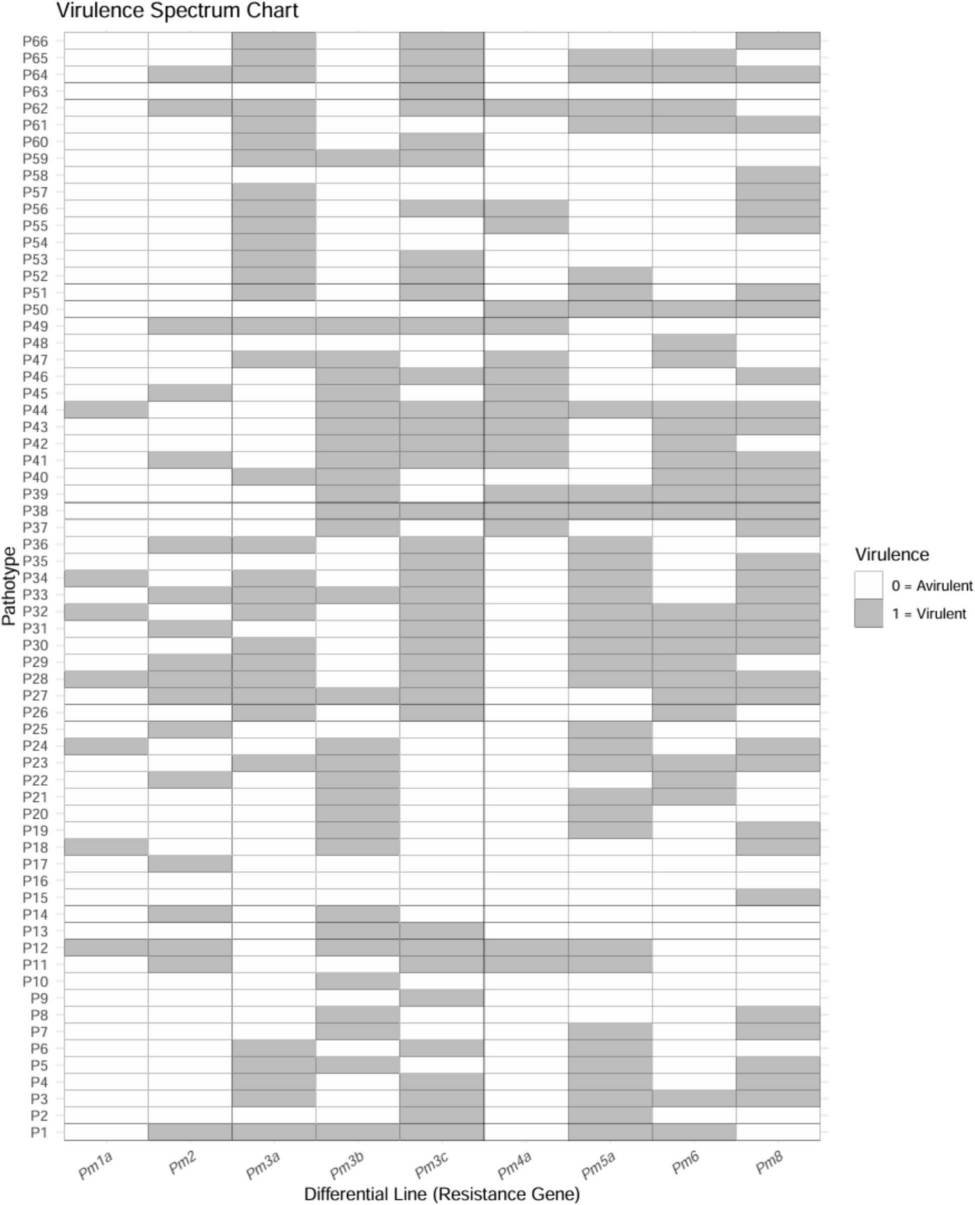
Infection response of differential lines to pathotypes (n = 66) of *B. graminis* f. sp. *tritici* from Himachal Pradesh during 1994–98. Differential lines are shown on the x-axis and pathotypes on the y-axis. White and gray indicate avirulence and virulence, respectively. Pathotypes P1-P15 are of ascosporic origin

Pathogenicity analysis of 215 *Bgt* isolates (155 conidial; 60 ascosporic) collected between 1994 and 1998, using nine differential lines, delineated 51 and 15 distinct pathotypes, respectively. Among the conidial isolates, pathotype P28 (from Kukumseri location (District Lahaul and Spiti) exhibited the broadest virulence spectrum, overcoming seven of the nine tested resistance genes (*Pm1a*, *Pm2*, *Pm3a*, *Pm3c*, *Pm5a*, *Pm6*, and *Pm8*). This was followed by several pathotypes (P27, P32, P33, P38, P41, P44, P62, and P64), virulent on six *Pm* genes, and others (P29, P30, P31, P34, P39, P43, and P49) virulent on five. In contrast, pathotype P20 (conidial), isolated from Joginder Nagar (District Mandi), was avirulent on all tested genes. The subsequent least virulent pathotypes (P21, P48, P54, P58, and P63), originating from Palampur and Simplan (District Kangra), each overcame only a single gene (Fig. 1; Table S1). Within the ascosporic isolates, pathotypes P1a and P12a (both from Kukumseri, District Lahaul and Spiti), displayed the highest virulence, each overcoming an average of six resistance genes (*Pm2*, *Pm3a*, *Pm3b*, *Pm3c*, *Pm5a*, and *Pm6* for pathotype P1a; *Pm1a*, *Pm2*, *Pm3b*, *Pm3c*, *Pm4a*, and *Pm5a* for pathotype P12a). Pathotype 3a, virulent against five genes, was the next most virulent (Fig. 1; Table S1).

### Virulence structure of *Bgt* populations (2015–2019)

**Fig. 2:**
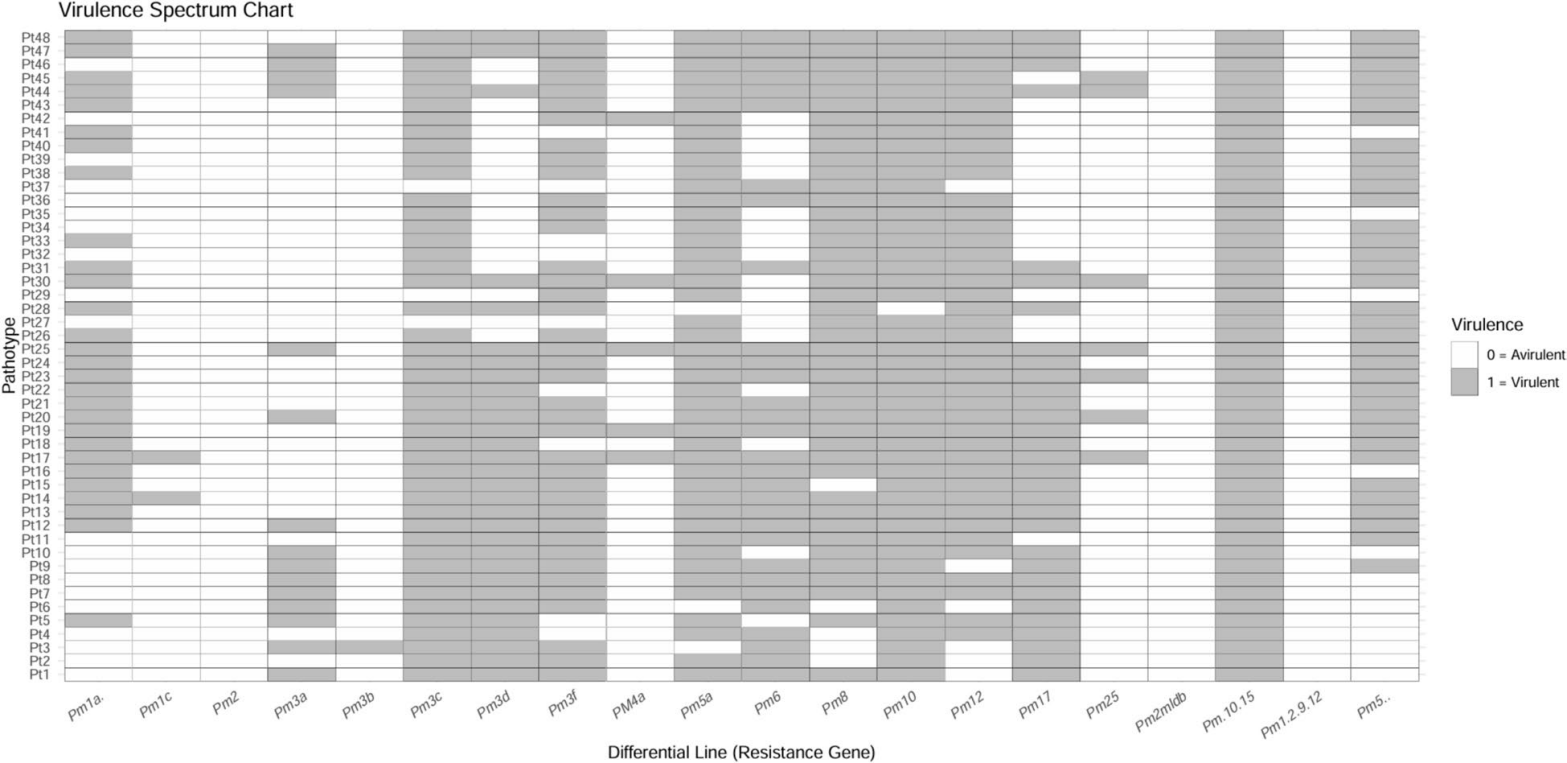
Infection response of differential lines to pathotypes (n = 48) of *B. graminis* f. sp. *tritici* from Himachal Pradesh during 2015–2019. Differential lines are shown on the x-axis and pathotypes on the y-axis. White and gray indicate avirulence and virulence, respectively. Pathotypes Pt26-Pt48 are of ascosporic origin

Evaluation of 70 *Bgt* isolates (45 conidial, 25 ascosporic) collected during 2015–2019 using 20 differential lines resolved 48 distinct pathotypes (25 conidial; 23 ascosporic). Conidial isolates exhibited a broad spectrum of virulence. Pathotypes Pt17 (Sarol, District Chamba) and Pt25 (Parour and Malan, District Kangra) were the most virulent, overcoming 15 of the 20 resistance genes (*Pm1a*, *Pm1c*, *Pm3a*, *Pm3c*, *Pm3d*, *Pm3f*, *Pm4a*, *Pm5a*, *Pm6*, *Pm8*, *Pm10*, *Pm12*, *Pm17*, *Pm25*, *Pm 10* + *15*, and *Pm5* + *?*)*.* These were followed by pathotypes Pt20 and Pt44 (virulent on 14 genes), and Pt12, Pt14, Pt19, and Pt23 (virulent on 13 genes). In contrast, pathotypes Pt2 (Palampur, District Kangra), Pt4, and Pt6 (both from Kukumseri, District Lahaul and Spiti) were the least virulent conidial pathotypes, showing susceptibility on only 8 genes (*Pm3a*, *Pm3c*, *Pm3d*, *Pm3f*, *Pm5a*, *Pm6*, *Pm10*, *Pm12*, *Pm17* and *Pm 10* + *15*). Pathotype Pt3 (Dalang Maidan) and Pt6 (Kukumseri), both of District Lahaul and Spiti, were virulent on 9 genes (Fig. 2; Table S2). Among ascosporic isolates, pathotype Pt44 (from district Lahaul and Spiti) displayed the highest virulence, overcoming 14 resistance genes. On average, conidial-derived pathotypes possessed a broader virulence spectrum (average virulence spectrum: 15 genes) than those derived from ascospores (Fig. 2; Table S2).

### Temporal shifts in virulence diversity and population differentiation

**Fig. 3:**
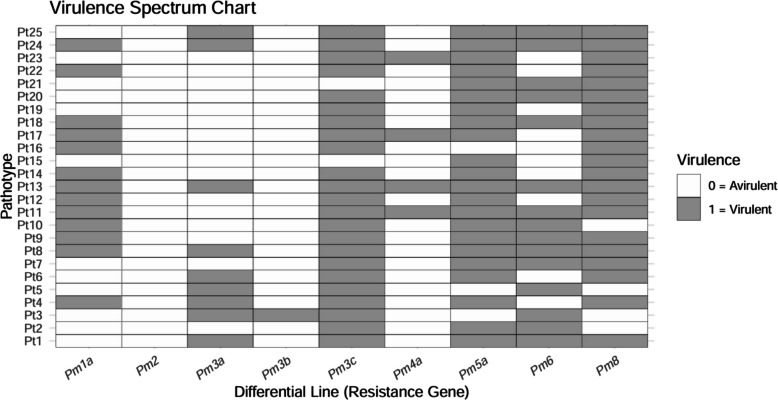
Infection response of common differential lines (*n* = 9) to pathotypes (*n* = 25) of *Blumeria graminis* f. sp. *tritici* from Himachal Pradesh during 2015–2019. Differential lines are shown on the x-axis and pathotypes on the y-axis. White and gray indicate avirulence and virulence, respectively. Pathotypes Pt14-Pt25 are of ascosporic origin

**Fig. 4:**
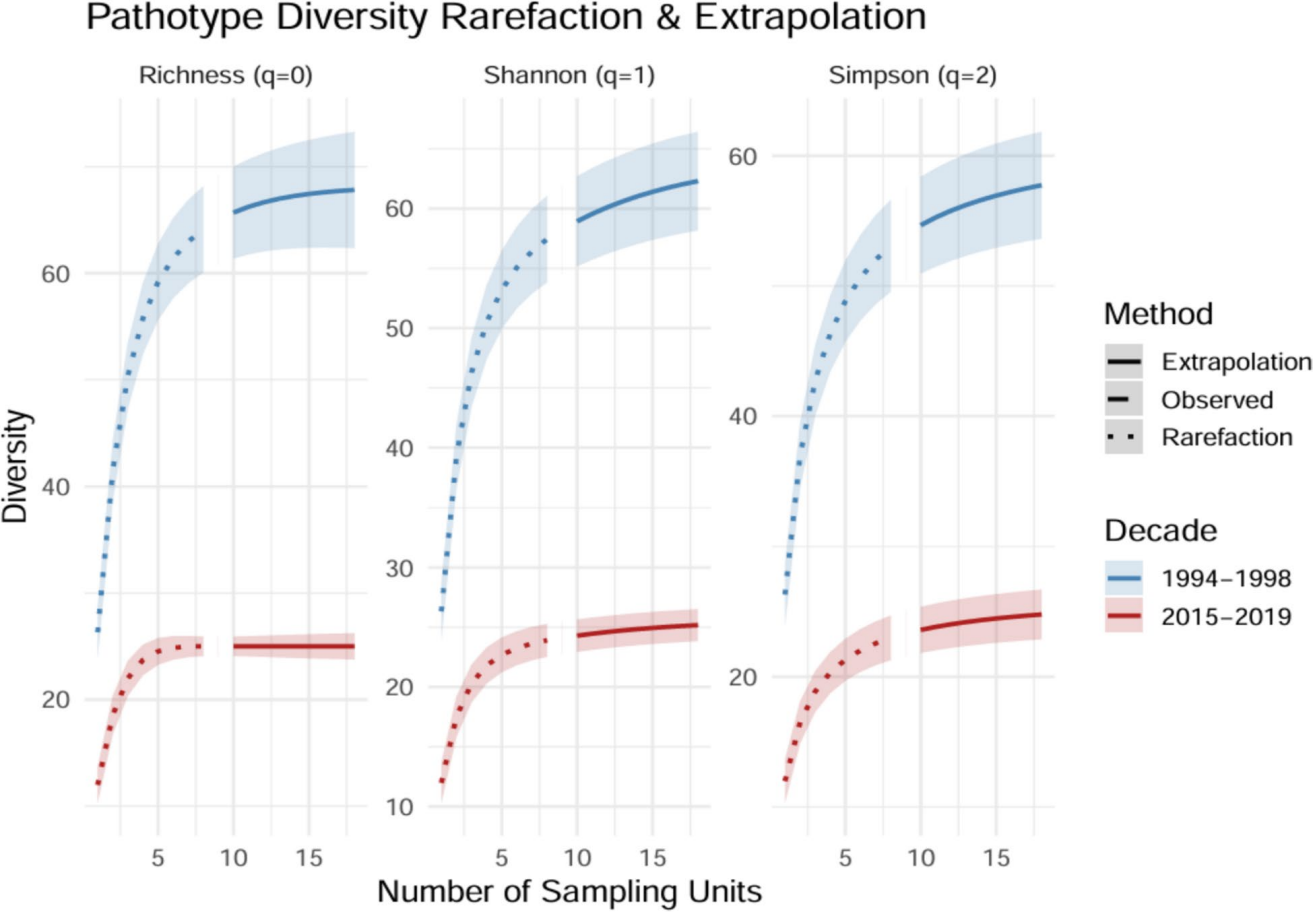
Refraction curves based on Hill numbers of orders (q = 0 {richness}), 1 {exponential of *Shannon’s* entropy index}, and 2 {inverse of *Simpson’s* concentration index}) were used to estimate the diversity of pathotypes *of B. graminis* f. sp. *tritici* from the study during 1994–98 (*n* = 66) and from 2015–19 (*n* = 25)

**Fig. 5:**
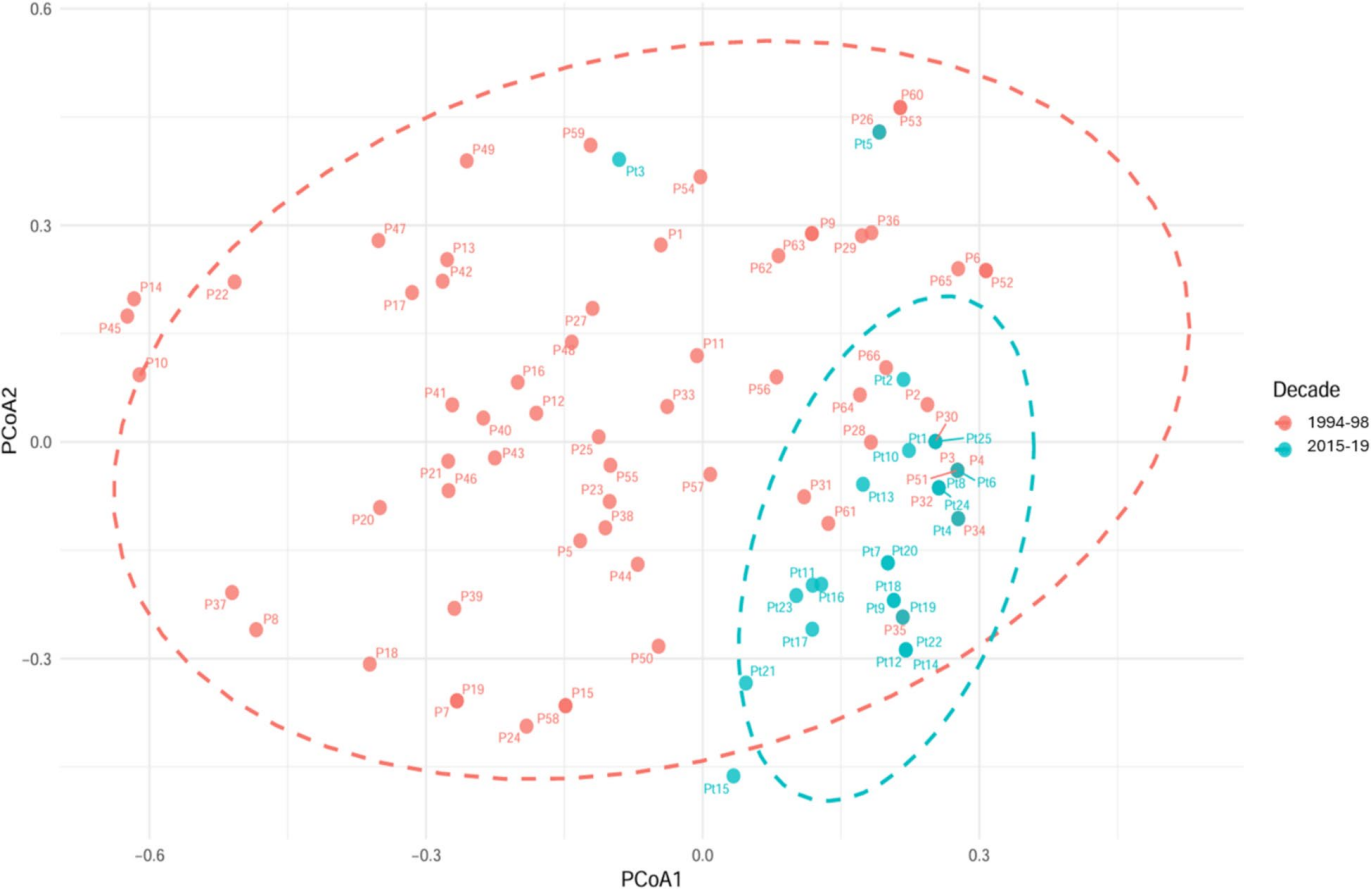
PCoA of *B. graminis* f. sp. *tritici* pathotypes collected in Himachal Pradesh during 1994–98 and 2015–19 based on their reaction to 9 common differentials

Comparative analysis on a common set of nine differential lines revealed significant temporal evolution in virulence structure (Figs. 1 and 3). According to the definition described in the materials and methods, the mean virulence complexity per isolate increased significantly from 3.92 (1994–1998) to 4.61 (2015–2019). Pathotype richness (expressed as the number of distinct pathotypes identified relative to the total number of isolates assayed) and evenness (quantifying the equitability of pathotype frequency distribution) were also higher in 2015–2019 population (0.316 and 0.820, respectively), compared to 1994–1998 population (0.306 and 0.756) (Table 1). Despite this increase in average virulence, overall pathogen population diversity declined. The 1994–1998 population exhibited greater diversity, with higher values for Simpson’s (*S*_*i*_ = 0.888), normalized Shannon’s (*S*_*h*_ = 3.085), and Kosman’s (*KW*_*m*_ = 0.420) indices compared to the 2015–2019 population (*S*_*i*_ = 0.864, *S*_*h*_ = 2.414, *KW*_*m*_ = 0.283). A concurrent decline in the Gleason index (*G*) further supported this temporal reduction in pathotype diversity (Table 1). These findings were supported by Hill number analysis, where non-overlapping confidence intervals for all diversity orders (*q* = *0–2*) confirmed a significant decrease in diversity in the 2015–2019 population (Fig. 4). Significant genetic differentiation between the two temporal populations was confirmed by all three applied distance metrics: Roger’s (*R*), Kosman’s (*KB*_*m*_), and Mean Character Difference (MCD) (Table 2). Permutational multivariate analysis of variance (PERMANOVA) validated that the differences in virulence structure were statistically significant (R^2^ = 0.082, *p* < *0.001*). Furthermore, a test of beta-dispersion indicated a significant reduction in multivariate variance within the 2015–2019 population (mean difference = 0.760, *p* < *0.001*) (Table 3). Principal coordinates analysis (PCoA) visually reinforced these results, revealing distinct clustering of pathotypes by decade. The first two PCoA axes explained 24.94% and 21.23% of the virulence variation, respectively (Fig. 5). Pathotypes from 1994–1998 showed greater dispersion, reflecting higher diversity, whereas those from 2015–2019 formed a tighter cluster, indicative of a more genetically homogeneous contemporary population (Fig. 5).

**Table 1:** Comparison of *B. graminis* f. sp. *tritici* population parameters during 1994–98 and 2015–19 on common differential lines (n = 9)

Parameters	Population	
	**1993–98**	**2015–19**
Number of isolates	215	70
Number of differentials	9	9
Number of pathotypes	66	25
Average virulence complexity per isolate	3.92	4.61
Average of relative virulence complexity	0.436	0.513
Richness (no. of pathotypes/number of isolates)	0.306	0.316
Evenness	0.756	0.820
Simpson’s index of diversity, *Si*	0.888	0.864
Shannon normalized index of diversity, *S*_*h*_	3.085	2.414
Kosman’s index of diversity, *KW*_*m*_	0.420	0.283
Gleason’s diversity index, G	10.91	4.237

**Table 2:** Genetic distance metrics (*Roger’s* distance (*R*), *Kosman’s* distance (*KBm*), and mean character difference (MCD)) between two *B. graminis* f. sp. *tritici* populations on common differential lines (n = 9)

Population	Rogers Distance (*R*)		Kosman’s Distance (*KB*_*m*_)		Mean Character Difference (MCD)	
	**1994–98**	**2015–19**	**1994–98**	**2015–19**	**1994–98**	**2015–19**
**1994–98**	0	0.808	0	0.059	0	0.250
**2015–19**	0.808	0	0.059	0	0.250	0

**Table 3:** Pairwise PERMANOVA and Beta dispersion comparisons of *B. graminis* f. sp. *tritici* populations over two decades (1994–98 and 2015–19)

**PERMANOVA**	**df**	**R**^**2**^	***F value***	***p*** **value**
Decade	1	0.082	7.951	< 0.001
Residual	89	0.917		
Total	90	1.000		
**Beta dispersion**	**df**	**Sum of squares**	***F value***	***p* value**
Decade	1	0.760	40.78	< 0.001
Residuals	89	1.659

### Temporal shifts in virulence gene associations

Comparative analysis of virulence co-occurrence for nine common *Pm* genes (*Pm1a*, *Pm2*, *Pm3a*, *Pm3b*, *Pm3c*, *Pm4a*, *Pm5a*, *Pm6*, *Pm8*) identified substantial evolutionary shifts between the two decades (Table 4). This shift was characterized by significant dissociations and the emergence of new virulence combinations. Most notably for the *Pm3b*-*Pm8* pair, which had a co-occurrence of −25.8%, in 1994–1998, was completely absent in the 2015–2019 population, representing a complete dissociation. Other significant dissociations included *Pm2*-*Pm3c* and *Pm3b*-*Pm3c* (each −19.7%), *Pm3b*-*Pm4a* (−18.2%) and *Pm2*-*Pm5* (−16.7%). Conversely the analysis revealed the strong emergence of new, positively associated virulence pairs. The largest increases in co-occurrence were observed for *Pm5a*-*Pm8* (+ 48.2%), *Pm3c*-*Pm8* (+ 47.2%), and *Pm3c*-*Pm5a* (+ 45.2%). Other pairs, such as *Pm1a*-*Pm3c* (+ 44.4%) and *Pm1a*-*Pm5a* (+ 38.9%), also showed strong, newly established positive linkages. The remaining gene pairs exhibited stable co-occurrence frequencies, suggesting maintained linkage within the pathogen population (Table 4).

**Table 4:** Virulence co-occurrence and pairwise association of *B. graminis* f. sp. *tritici* populations on common differential lines (n = 9) during 1994–98 and 2015–19

Gene A	Gene B	Cooccurrence in 1994–98 (%)	Cooccurrence in 2015–19 (%)	Association (%)	Interpretation
*Pm1a*	*Pm2*	3	0	−3	Stable association
*Pm1a*	*Pm3a*	4.5	16	11.5	Stable
*Pm1a*	*Pm3b*	6.1	0	−6.1	Stable
*Pm1a*	*Pm3c*	7.6	52	44.4	New association
*Pm1a*	*Pm4a*	3	12	9	Stable
*Pm1a*	*Pm5a*	9.1	48	38.9	New association
*Pm1a*	*Pm6*	4.5	28	23.5	New association
*Pm1a*	*Pm8*	9.1	48	38.9	New association
*Pm2*	*Pm3a*	13.6	0	−13.6	Stable
*Pm2*	*Pm3b*	13.6	0	−13.6	Stable
*Pm2*	*Pm3c*	19.7	0	−19.7	Dissociation
*Pm2*	*Pm4a*	9.1	0	−9.1	Stable
*Pm2*	*Pm5a*	16.7	0	−16.7	Dissociation
*Pm2*	*Pm6*	13.6	0	−13.6	Stable
*Pm2*	*Pm8*	9.1	0	−9.1	Stable
*Pm3a*	*Pm3b*	13.6	4	−9.6	Stable
*Pm3a*	*Pm3c*	36.4	36	−0.4	Stable
*Pm3a*	*Pm4a*	7.6	4	−3.6	Stable
*Pm3a*	*Pm5a*	28.8	28	−0.8	Stable
*Pm3a*	*Pm6*	22.7	28	5.3	Stable
*Pm3a*	*Pm8*	27.3	28	0.7	Stable
*Pm3b*	*Pm3c*	19.7	4	−15.7	Stable
*Pm3b*	*Pm4a*	18.2	0	−18.2	Dissociation
*Pm3b*	*Pm5a*	19.7	0	−19.7	Dissociation
*Pm3b*	*Pm6*	19.7	4	−15.7	Stable
*Pm3b*	*Pm8*	25.8	0	−25.8	Dissociation
*Pm3c*	*Pm4a*	16.7	16	−0.7	Stable
*Pm3c*	*Pm5a*	34.8	80	45.2	New association
*Pm3c*	*Pm6*	25.8	56	30.2	New association
*Pm3c*	*Pm8*	28.8	76	47.2	New association
*Pm4a*	*Pm5a*	10.6	16	5.4	Stable
*Pm4a*	*Pm6*	13.6	8	−5.6	Stable
*Pm4a*	*Pm8*	15.2	16	0.8	Stable
*Pm5a*	*Pm6*	25.8	52	26.2	New association
*Pm5a*	*Pm8*	31.8	80	48.2	New association
*Pm6*	*Pm8*	24.2	44	19.8	New association

### Temporal efficacy of *Pm* genes and virulence pressure

**Fig. 6:**
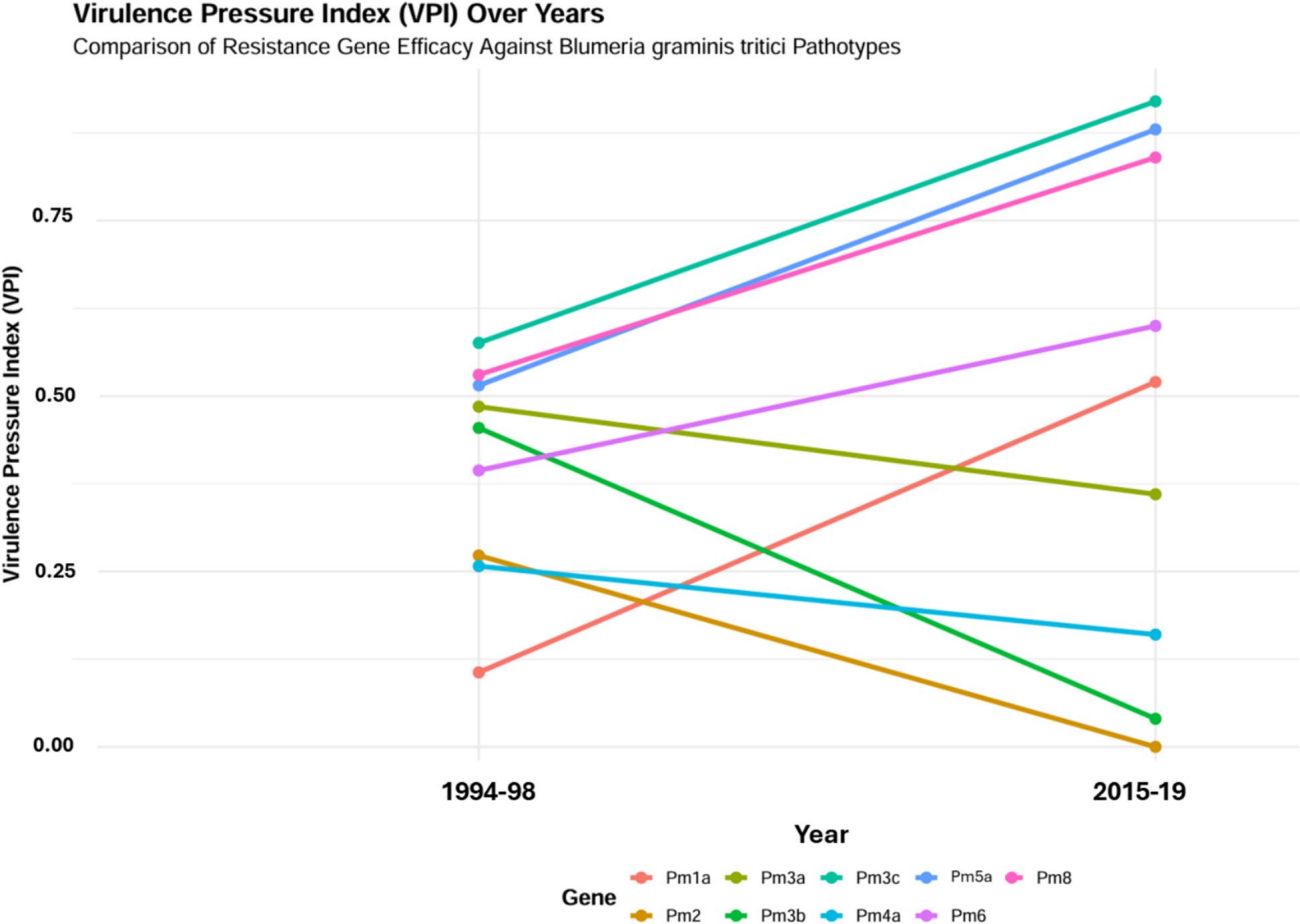
Temporal changes in resistant gene efficacy against *B. graminis* f. sp. *tritici* populations during 1994–98 and 2015–19

**Fig. 7:**
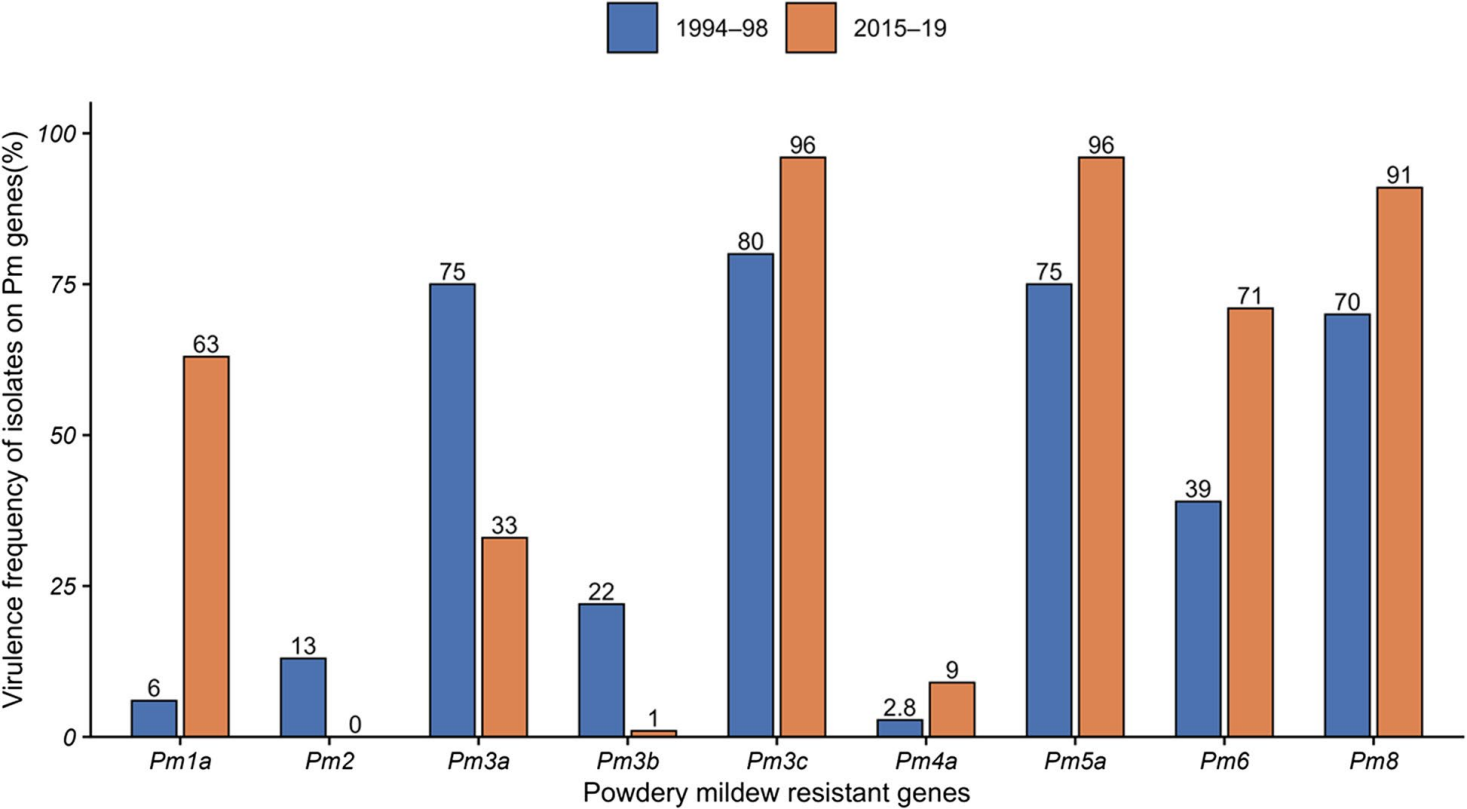
Virulence frequency of *B. graminis* f. sp. *tritici* isolates on common differential lines during 1994–98 and 2015–19

Analysis over two decades revealed significant temporal shifts in virulence frequency for several key resistance genes (Fig. 6). Fisher’s exact test identified statistically significant changes (*p* < *0.05*) for *Pm1a*, *Pm2*, *Pm3b*, *Pm3c*, *Pm5a*, and *Pm8*, whereas the virulence frequencies for *Pm3a*, *Pm4a*, and *Pm6* remained unchanged (Table 5). The Virulence Pressure Index (VPI) has defined as the proportion of *Bgt* pathotypes virulent to given *Pm* gene relative to the total pathotypes tested over time. The most notable VPI increase was observed for *Pm1a* indicating intensified selection for compatible pathotypes and a heightened risk of resistance erosion. Similarly, *Pm3c*, *Pm5a*, and *Pm8* maintained high VPI values across both periods. Although the shift in virulence frequency for *Pm6* was not statistically significant, its VPI increased from 0.39 to 0.69, indicating a concerning trend toward potential resistance breakdown (Fig. 6). Linear regression analysis of resistance efficacy over time statistically corroborated these patterns of increasing virulence pressure. Genes *Pm1a*, *Pm3c*, *Pm5a*, *Pm6*, and *Pm8* exhibited significant negative slopes (β₁ = −0.414, −0.344, −0.365, −0.206, and −0.310, respectively), confirming a significant decline in their protective efficacy (Table 5). In contrast, genes *Pm2*, *Pm3b*, and *Pm4a* demonstrated consistently low VPI and stable efficacy, indicating their inherent durability against the prevailing *Bgt* populations (Figs. 6, 7, Table 5).

**Table 5:** Durability classification of common resistance genes assessed during 1994–98 and 2015–19 based on the slope of efficacy trends over time

Genes	Slope	Type	Fisher Test	VPI^*^	Risk
*Pm1a*	−0.414	*Broken*	0.0001	0.219	Low
*Pm2*	*0.273*	*Durable*	0.0023	0.197	Low
*Pm3a*	*0.125*	*Durable*	0.0587	0.451	Medium
*Pm3b*	*0.415*	*Durable*	0.0001	0.341	Medium
*Pm3c*	*−0.344*	*Broken*	0.0022	0.670	High
*Pm4a*	*0.097*	*Durable*	0.4107	0.231	Low
*Pm5a*	*−0.365*	*Broken*	0.0015	0.615	High
*Pm6*	*−0.206*	*Broken*	0.0610	0.451	Medium
*Pm8*	*−0.310*	*Broken*	0.0078	0.615	High

Negative slopes indicate declining efficacy. Genes were classified into two durability categories: Durable (slope > −0.01) and Broken (slope ≤ −0.05). A *p* < *0.05* indicates a significant change in virulence for that gene over time

^*^Virulence Pressure Index (VPI) represents the average across the two study periods (1994–98 and 2015–19)

## Discussion

The high evolutionary potential of *Bgt*, driven by both sexual recombination and asexual mutation, continuously challenges the durability of race-specific resistance genes in wheat (Pretorius et al. 2000). The large-scale monoculture of resistant varieties exerts intense selection pressure, often resulting in the rapid emergence of matching virulences and the subsequent resistance breakdown (Parks et al. 2008; Paul et al. 2000; Basandrai and Basandrai 2017; Wu et al. 2019; Xue et al. 2021; Basandrai et al. 2023). Our longitudinal study, comparing virulence surveys conducted over two decades (1994–1998 and 2015–2019) in the epidemiologically significant Northwestern Himalayan region, offers a unique perspective on this co-evolutionary arms race. The findings reveal significant shifts in pathogen population structure, the erosion of key resistance genes, and the emergence of new virulence associations, thereby delivering critical insights for future resistance management.

Our analysis revealed a paradoxical but significant trend, although the mean virulence complexity per isolate increased in the contemporary population, indicating enhanced pathogenic capability, the overall genetic diversity of the *Bgt* population decreased (Laloševic´ et al. 2022). This was supported by a decline in key diversity indices (Simpson’s, Shannon’s, and Kosman’s) and confirmed by non-overlapping confidence intervals of Hill numbers. This pattern is indicative of selective sweep, whereby a few highly virulent, genetically similar pathotypes have risen to dominance, likely driven by the widespread cultivation of varieties with a narrow genetic base. This finding aligns with global reports on the clonal expansion of fit, virulent lineages in powdery mildew populations (Wu et al. 2019; Lalošević et al. 2022). The significant differentiation between the two temporal populations, validated by Roger’s and Kosman’s distances and PERMANOVA, confirms a fundamental restructuring of the pathogen population over time (Dreiseitl et al. 2006; Wang et al. 2023). A critical aspect of this restructuring is the ongoing reshuffling of virulence genes. We observed both the dissociation of formerly linked virulences (*Pm3b*-*Pm8*) and the formation of potent new associations (*Pm5a*-*Pm8*, *Pm3c*-*Pm8*). The breakdown of established linkages may reflect a fitness cost associated with maintaining unnecessary virulence factors, particularly if the corresponding resistance genes have been withdrawn from deployment (Paul et al. 2000; Kanwar 1993). Conversely, the emergence of new virulence clusters represents a clear adaptive response, potentially enabling the pathogen to overcome pyramided resistance genes. From a breeding perspective, dissociated gene pairs represent promising candidates for gene pyramiding, as the pathogen may struggle to recombine these virulences simultaneously.

The temporal analysis of the VPI and resistance efficacy provides a direct assessment of resistance gene durability. Our data clearly indicates the erosion of several widely deployed genes. The rapid increase in VPI for *Pm1a* and *Pm6*, coupled with consistently high pressure on *Pm3c*, *Pm5a*, and *Pm8*, is further evidenced by significant negative slopes in their efficacy over time. The widespread defeat of *Pm3c*, *Pm5a*, and *Pm8* in Himachal Pradesh is directly linked to their extensive historical deployment. These genes were often inadvertently introgressed into popular wheat varieties due to their close linkage with leaf rust (*Lr26*) and yellow rust (*Yr9*) resistance genes (Basandrai et al. 2016). The large-scale cultivation of varieties carrying the *Pm8/Lr26/Yr9* gene complex (PBW-343, HS-240, HPW-284, HD-2967, DPW 50, HS 507, HS 542 and HS 562) across the NWPZ and NHZ precipitated a dramatic increase in virulence frequency against *Pm8*. This frequency escalated from < 10% in the early 1990 s (Sharma et al. 1990; Sharma and Singh 1990a, 1990b) to > 50% within a decade (Paul et al. 2000) and exceeds 80% in the present study. This pattern was reinforced over three decades by the national cultivation of popular varieties derived from progenitors like Kalyansona and Sonora (*Pm3c*), many carrying *Lr26/Yr9* (e.g., PB343, HPW-42, HS 240, HS-277, HUW 206, K 8804, HW 318, MACS 2496, VL 738, UP 2338, WH-542, VL 829, VL 907) or *Lr23* (e.g., HPW-251, HD-2967, WH 1021, K 8027, GW 322, HD 2285, HS 295, PBW 154) (Bhardwaj 2011; Anonymous 2014). The prolonged and widespread deployment of these varieties exerted intense directional selection on the *Bgt* population, leading to the near fixation of virulence against the linked *Pm3c*, *Pm5a*, and *Pm8* genes across India (Bahadur and Aggarwal 1997; Paul et al. 2000; Basandrai et al. 2003, 2016; Mehta et al. 2024, 2025). Basandrai et al. (2003) documented this shift, noting prevalent virulence on *Pm3a*, *Pm3c*, *Pm3e*, *Pm3f*, *Pm5*, and *Pm6*, while virulence on *Pm1*, *Pm2*, *Pm4a*, and *Pm2* + *6* remained rare. A parallel trend is evident for *Pm1a* and *Pm6*, whose increased virulence in northern India is directly linked to their deployment in widely grown cultivars such as HS-542, DBW-179, WH-1181, HPBW 01, and DDK-1051 (Mehta et al. 2025). The incorporation of these genes into the genetic background of susceptible host populations has provided a strong selective advantage for pathogen genotypes capable of overcoming them, driving the observed increase in corresponding virulence frequencies.

In stark contrast, the genes *Pm2*, *Pm3b*, and *Pm4a* have demonstrated remarkable durability, maintaining low VPI and stable efficacy for over two decades. The persistent effectiveness of *Pm2* and *Pm4a* against Himalayan *Bgt* populations, also noted in studies from Egypt and Hungary (Abdelrhim et al. 2018; Szunics et al. 2001), marks them as prime candidates for future breeding programs. The combination of *Pm2* and *Pm4a* has proven highly robust, suggesting synergistic effects that enhance durability (Moustafa et al. 2016; Abdelrhim et al. 2018). Earlier studies from Himachal Pradesh and Punjab (India) also recorded very low virulence on these genes (Sharma and Singh 1990a, 1990b; Sharma et al. 1990; Paul et al. 2000).

## Conclusion

In conclusion, this two-decade study underscores the relentless adaptability of *Bgt* and the inherent vulnerability of monogenic resistance. The observed genetic shifts towards higher individual virulence but lower population diversity highlight a pathogen population undergoing adaptive refinement. The documented erosion of *Pm1a*, *Pm6*, and *Pm8* serves as a critical warning against over-reliance on single, widely deployed genes. Conversely, the identified durable resistance sources, particularly *Pm2* and *Pm4a*, offer a clear strategic path forward. Additionally, the effectiveness of genes *Pm1c, Pm3b, Pm2* + *Mld* and *Pm1* + *2* + *9* + *12* during 2015–2019 suggests their potential utility in breeding programs. We therefore advocate for a strategic shift toward pyramiding these validated, durable genes with other effective sources, guided by continuous, region-specific virulence monitoring. Such a dynamic and diversified approach is essential to develop resilient wheat varieties and ensure long-term, sustainable management of powdery mildew in the Himalayas and beyond.

## Materials and methods

### Study overview and temporal framework

This study was conducted to characterize the virulence structure of *Bgt* populations collected between 2015 and 2019 and to analyze its time-series dynamism by comparing it with virulence dataset studied from the period of 1994–1998.

### Pathogen isolate collection

**Fig. 8:**
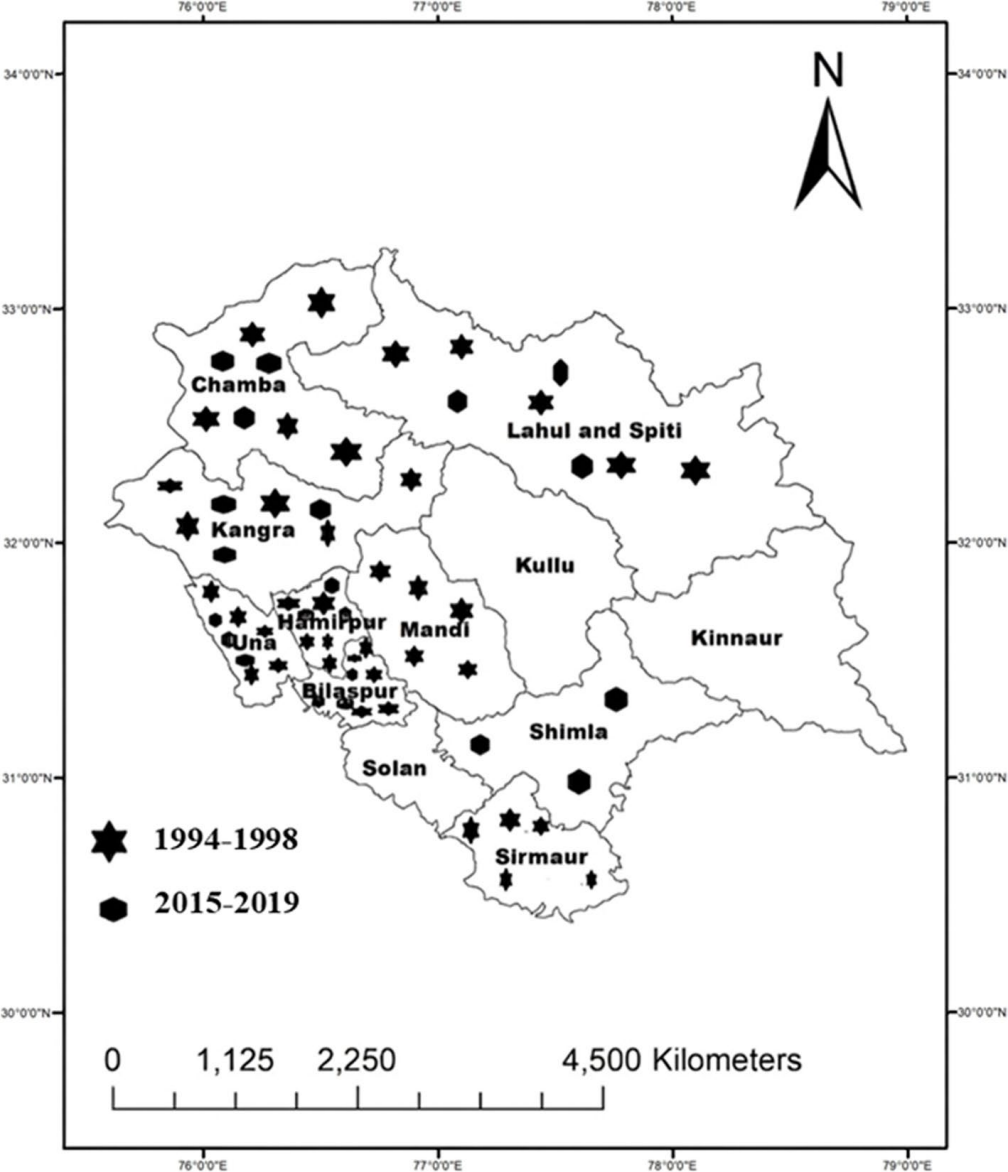
Geographic distribution of sampling sites in different agro-climatic regions of Himachal Pradesh for the collection of wheat powdery mildew samples during 1994–1998 and 2015–19

A total of 285 *Bgt* isolates were analyzed across the two study periods. The contemporary population (2015–2019) consisted of 70 single-colony isolates (45 conidial; 25 ascosporic), while the historical population (1994–1998) comprised 215 isolates (155 conidial; 60 ascosporic). Isolates were collected from major wheat-growing, agro-climatic zones within the Northwestern Himalayan state of Himachal Pradesh, India. Sampling locations included the districts of Lahaul and Spiti (Dalang Maidan and Kukumseri), Kangra, Bilaspur, Una, Chamba, Shimla, and Hamirpur (Fig. 8; Table S3 and S4). Ascosporic isolates were obtained from chasmothecia (cleistothecia) sampled in the dry temperate zone of Dalang Maidan and Kukumseri. These fruiting bodies were matured in the laboratory to induce ascospores release for isolate establishment.

### Conidial isolates

**Fig. 9:**
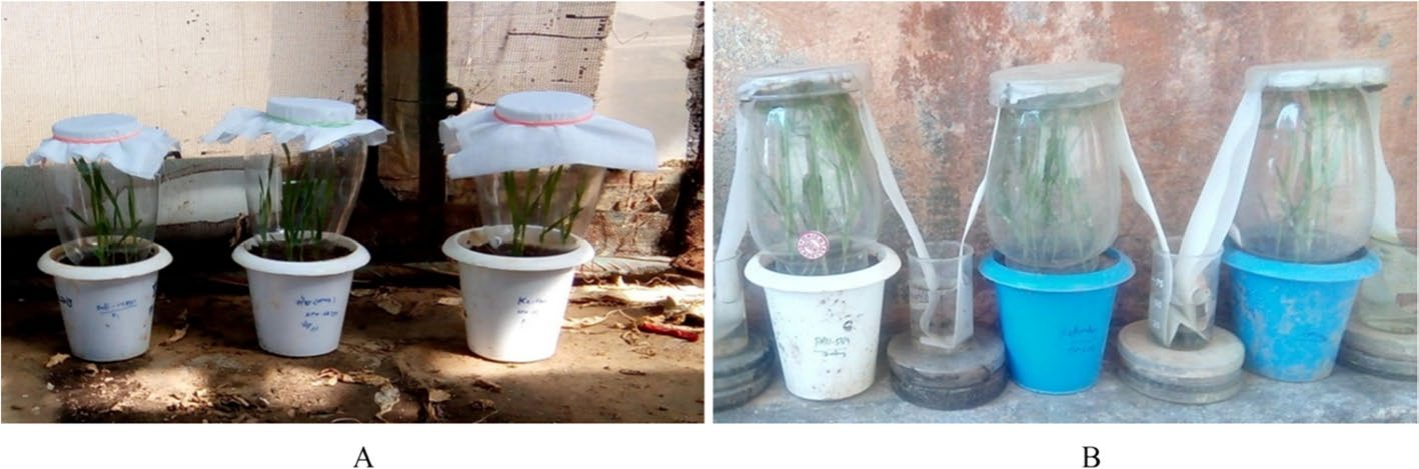
Methods for isolation and maintenance of (**a**) conidial and (**b**) ascosporic isolates of *B. graminis* f. sp. *tritici*

Leaves or ear heads exhibiting powdery mildew symptoms were collected from the field. In laboratory, leaf segments with actively sporulating colonies were placed on Petri plates containing 2% water agar. Under aseptic conditions, conidia from distinct colonies were individually transferred onto primary leaves of fourteen-day-old seedlings of the susceptible cultivars 'Agra Local' or 'Lehmi' using a sterilized camel-hairbrush (Fig. 9A). Each *Bgt* isolate was multiplied and maintained in spore-proof isolation chambers, with inoculum renewed on fresh susceptible seedlings every 15–20 days. The total surviving and maintained conidial isolates numbered 155 for the 1994–1998 period and 45 for the 2015–2019 period.

### Ascosporic isolates

Leaves bearing cleistothecia were collected from the dry temperate zone of Himachal Pradesh (Dalang Maidan, Keylong, and Kukumseri in Lahaul and Spiti district). Individual cleistothecia were excised and placed on moist blotter paper within watch glasses. These watch glasses were then inverted over glass chimneys enclosing fourteen-day-old seedlings of the susceptible cultivars (Lehmi and Agra Local) to capture discarded ascospores. The cleistothecia were kept hydrated via blotter paper wicks. Ascospore release was monitored from the fourth day onward (Fig. 9B). Fungal colony development was observed 7 days post ascospore discharge and isolated colonies were subsequently purified via conidia transfer to fresh seedlings for mass production. This process yielded 60 and 25 ascosporic isolates for the 1994–1998 and 2015–2019 periods, respectively.

### Virulence phenotyping and pathogenic variation

#### Differential hosts

The virulence spectrum of the *Bgt* isolates was assessed using a set of international powdery mildew differential lines (*Pm* lines). These lines, comprising near-isogenic lines (NILs) in the 'Chancellor' background and resistant cultivars, were sourced from the Indian Institute of Wheat and Barley Research (IIWBR), Karnal, the International Maize and Wheat Improvement Center (CIMMYT), Mexico, and Punjab Agricultural University (PAU), Ludhiana. A detailed list of the differential lines, their corresponding *Pm* genes, and their use across study periods is provided in Table 6.

**Table 6:** Differential lines used for phenotyping virulence of powdery mildew (*B. graminis* f. sp. *tritici*) isolates (2015–2019 and 1994–1998)

Sr. no	Differential lines/cultivars	*Pm* genes
1	Near isogenic line (Axminister x Cc^8^)	Pm1a^*^
2	Whmn	*Pm1c*
3	Near isogenic line (Ulka x Cc^8^)	Pm2^*^
4	Near isogenic line (Asosan x Cc^8^)	Pm3a^*^
5	Chul (Chul x Cc^8^)	Pm3b^*^
6	Near isogenic line (Sonora x Cc^8^)	Pm3c^*^
7	Kolibri	*Pm3d*
8	Michigan Amber x Cc^8^ (CLTR 15888)	*Pm3f*
9	Khapli	Pm4a^*^
10	Hope	Pm5a^*^
11	Timgalen	Pm6^*^
12	Kavkaz	Pm8^*^
13	Norin	*Pm10*
14	Wembley	*Pm12*
15	Amigo	*Pm17*
16	NC96BGTA5	*Pm 25*
17	Maris dove	*Pm2* + *Mld*
18	Chancellor	*Pm 10* + *15*
19	Normandie	*Pm1* + *2* + *9* + *12*
20	Talent	*Pm5* + *?*
21	HPW 155/Lehmi/Agra Local	Susceptible check

^*^Common differential lines used in both the 1994–98 and 2015–2019 studies

#### Seedling assay, inoculation, and disease assessment

**Fig. 10:**
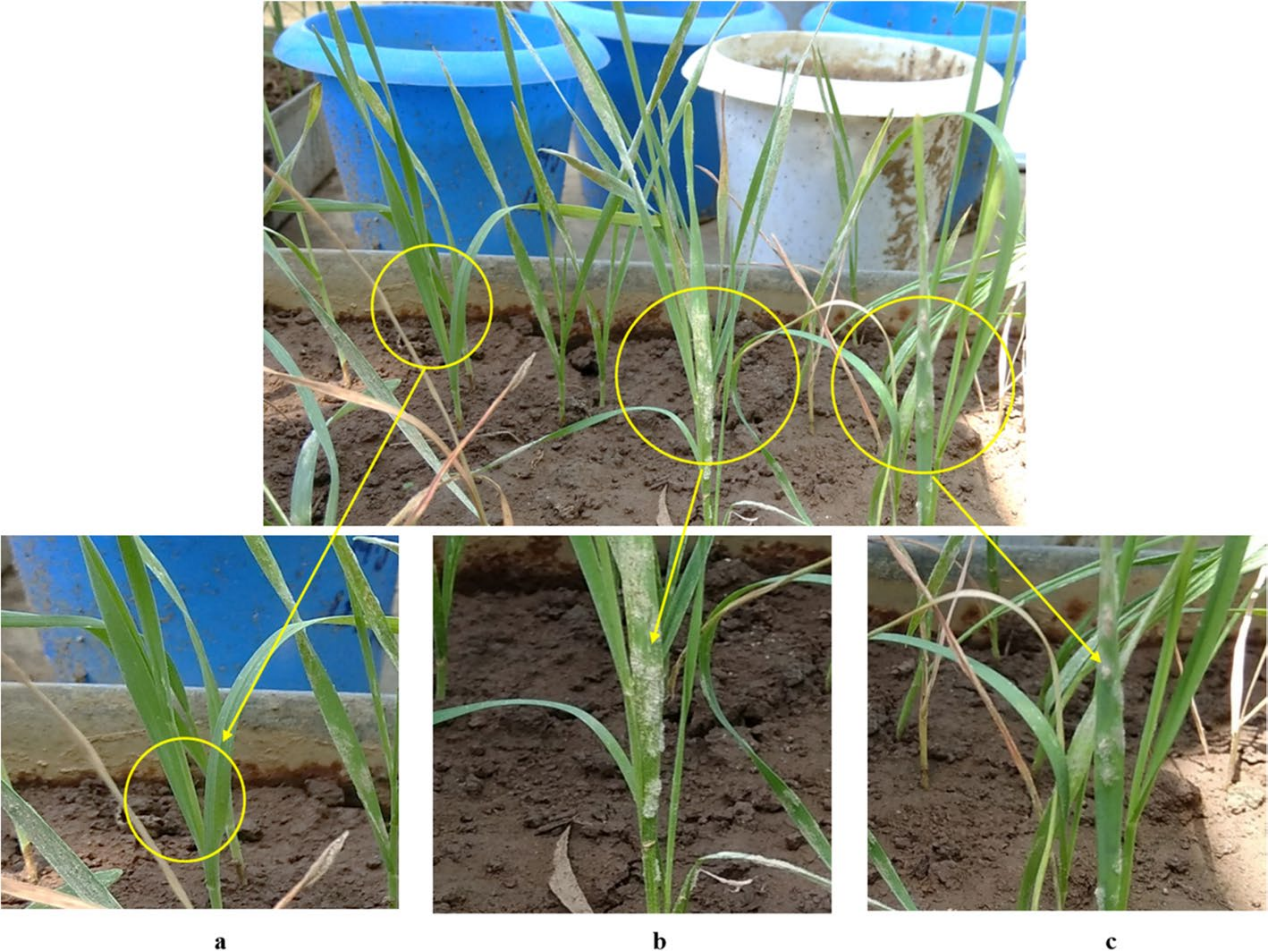
Representative infection types of *B. graminis* f. sp. *tritici* on differential lines; (**a**-**b**) resistant reactions (free from disease), and (**c**) susceptible reaction (abundant sporulation)

Seedlings of the differential lines and susceptible checks (Agra Local and Lehmi) were grown, inoculated, and incubated according to established protocols (Basandrai et al. 2016). Ten days post-inoculation, the infection type (IT) on each differential was recorded using the modified ‘0–4’ scale (Smith and Blair 1950). Isolates eliciting ITs of 0–2 were considered avirulent, while isolates producing ITs of 3–4 (Fig. 10) were classified as virulent (Si et al. 1987). For subsequent quantitative analyses, host reactions were coded binarily: resistant reactions (IT 0–2) as ‘0’ and susceptible reactions (IT 3–4) as ‘1’ (Wu et al. 2019). This binary matrix was used to construct a virulence spectrum chart and for all downstream statistical computations.

### Statistical analysis

Comparative virulence analysis was conducted using a core set of nine differential lines (*Pm* genes) phenotyped consistently across both study periods (1994–1998 and 2015–2019). Key population parameters were calculated, including virulence frequency, virulence complexity, relative virulence complexity, pathotype number, richness, and evenness. Virulence frequency for a specific *Pm* gene was defined as the proportion of isolates virulent on the corresponding differential line. For each isolate, virulence complexity was calculated as the number of susceptible differentials and thus ranged from 1 to 20. The corresponding relative virulence complexity values ranged from 0 to 1 (Laloševic´ et al. 2022). This relative measure was derived as the virulence complexity per differential line (Kosman 2003), with mean values subsequently calculated per isolate (Dreiseitl et al. 2006). Pathotype richness was expressed as the number of distinct pathotypes identified relative to the total number of isolates assayed. Pathotype evenness, quantifying the equitability of pathotype frequency distribution, was also calculated, with higher values indicating a more balanced distribution. To assess temporal changes in population structure, diversity and divergence metrics were employed. Pathotype based diversity was quantified using the normalized Shannon’s index (*S*_*h*_) and the Simpson’s index (*S*_*i*_), while divergence was measured by Roger’s distance (*R*). Furthermore, comprehensive genetic diversity and pairwise distance were evaluated using Kosman’s diversity (*KW*_*m*_) and distance (*KB*_*m*_) indices, which integrate both pathotype identity and multi-locus virulence data (Kosman et al. 2008). All analyses for descriptive parameters and these diversity indices were performed using the Virulence Analysis Tool (VAT) software (Kosman et al. 2008). For a robust, multi-dimensional comparison of pathotype diversity, Hill numbers of three orders (*q*) were calculated: *q* = 0 (equivalent to species richness), *q* = 1 (exponential of Shannon’s entropy index), and *q* = 2 (inverse of Simpson’s concentration index). Confidence intervals were generated for these estimates to enable statistical comparison between the two temporal populations (Chao et al. 2014; Batista et al. 2023). This analysis was conducted using the *iNEXT* package in R Studio (v4.0.1).

### Analysis of virulence associations and population shifts

#### Virulence dissociation and co-occurrence

Temporal shifts in virulence associations among the nine common *Pm* genes were analyzed. For each population (1994–1998 and 2015–2019), a binary virulence matrix was constructed, coding avirulent (resistant) reactions as ‘0’ and virulent (susceptible) reactions as ‘1’. Pairwise virulence co-occurrence for all gene combinations was then quantified as the percentage of pathotypes virulent on both genes A and B relative to the total number of pathotypes. To identify evolutionary shifts in association, the change in co-occurrence percentage for each gene pair between periods was calculated as: Change Co-occurrence (%) = Co-occurrence (2015–2019)—Co-occurrence (1994–1998). Based on this metric, gene pairs were classified as follows: those with a negative change value were categorized as dissociating, those with a value near zero as stable, and those with a positive change value as newly associated.

#### Multivariate statistical analyses

Multivariate analyses were performed to evaluate overarching differences in virulence structures between the two temporal populations. A Principal Coordinates Analysis (PCoA) was performed based on a Jaccard distance matrix to visualize the overall distribution and clustering of pathotypes. The homogeneity of multivariate dispersions (variance) with each population was tested using a beta-dispersion test. To statistically test for differences in the centroids of the virulence profiles, a Permutational Multivariate Analysis of Variance (PERMANOVA) with 999 permutations was conducted using the ‘adonis’ function to test significant differences in the centroids of the virulence profiles between the two time periods. Both multivariate analyses were performed using the *vegan* package (Oksanen et al. 2020), with visualizations generated using *ggplot2* (Wickham 2016).

#### Virulence pressure and resistance durability

The temporal durability of resistance genes and the selection pressure of pathogen were statistically evaluated. Virulence Pressure Index (VPI) was calculated to estimate the selection pressure exerted by the pathogen population on each gene over time. It was calculated by dividing the number of virulent pathotypes on a particular gene with total number of pathotypes tested during the study period. The values of VPI ranged from 0 (no virulence) to 1 (complete virulence). Concurrently, the durability of each gene was assessed by fitting a linear regression model with the mean efficacy score of the gene as the response variable and time as the predictor. The regression slope served as the durability indicator: genes with a non-significant or positive slope were categorized as 'Durable', while those with a significant negative slope were categorized as 'Broken'.

## Supplementary Information

Supplementary Material 1.

## Data Availability

Data will made available on reasonable requests.

## Abbreviations

*Bgt*: *Blumeria graminis* F. sp. *tritici*
*Sh*: Shannon normalized index of diversity
*Si*: *Simpson’s* Index of diversity
*R*: Roger’s distance
*KWm*: *Kosman’s* Index of diversity
*KBm*: *Kosman’s* Distance
VAT: Virulence analysis tool
*G*: *Gleason’s* Diversity index
MCD: Mean character distance
NILs: Near-isogenic lines
IT: Infection type
PAU: Punjab Agricultural University, Ludhiana
IIWBR: Indian Institute of Wheat and Barley Research, Karnal
CIMMYT: International Maize and Wheat Improvement Center, Mexico
PERMANOVA: Permutational Multivariate Analysis of Variance
PCoA: Principal Coordinates Analysis
VPI: Virulence Pressure Index
