# Supplementary material for: Deciphering a pathogen’s evolution: a two-decade longitudinal study reveals virulence shifts and identifies durable Pm genes against Himalayan Blumeria graminis f. sp. tritici populations
Source: Stress Biol. 2026 May 9;6(1):37. doi: 10.1007/s44154-026-00299-0 (PMC13157376; doi:10.1007/s44154-026-00299-0)
Supplement: Supplementary file 1 — Supplementary Material 1. [file 44154_2026_299_MOESM1_ESM.docx]

**Table S1: Reaction of isolates of *Blumeria graminis tritici* on differential lines carrying known gene(s) for powdery mildew resistance (*Pm* genes) during 1994-98.**

| **Isolates** | ***Pm1a*** | ***Pm2*** | ***Pm3a*** | ***Pm3b*** | ***Pzm3c*** | ***Pm4a*** | ***Pm5a*** | ***Pm6*** | ***Pm8*** | **Agra Local** | **Pathotypes** |
| --- | --- | --- | --- | --- | --- | --- | --- | --- | --- | --- | --- |
| 1, 55, 56, 58, 59 | R | S | S | S | S | R | S | S | R | S | 1^a^ |
| 2 | R | R | R | R | S | R | S | R | R | S | 2^a^ |
| 3, 11, 13, 20, 31, 42, 43, 47, 52, 54, 57, 4, 5, 6, 9, 12, 15, 27, 29, 37, 38, 39, 40, 41, 44, 47, 51, 29, 60, 59#, 65# | R | R | S | R | S | R | S | S | S | S | 3^a^ |
| 14, 18 | R | R | S | R | S | R | S | R | S | S | 4^a^ |
| 16, 36 | R | R | S | S | R | R | S | R | S | S | 5^a^ |
| 17 | R | R | S | R | S | R | S | R | R | S | 6^a^ |
| 23, 83, 97 | R | R | R | S | R | R | S | R | S | S | 7^a^ |
| 26 | R | R | R | S | R | R | R | R | S | S | 8^a^ |
| 28 | R | R | R | R | S | R | R | R | R | S | 9^a^ |
| 29, 55, 63 | R | R | R | S | R | R | R | R | R | S | 10^a^ |
| 30 | R | S | R | R | S | S | S | R | R | S | 11^a^ |
| 31 | S | S | R | S | S | S | S | R | R | S | 12^a^ |
| 32 | R | R | R | S | S | R | R | R | R | S | 13^a^ |
| 33 | R | S | R | S | R | R | R | R | R | S | 14^a^ |
| 35 | R | R | R | R | R | R | R | R | S | S | 15^a^ |
| **34, 38** | R | R | R | R | R | R | R | R | R | S | 16 |
| **40** | R | S | R | R | R | R | R | R | R | S | 17 |
| **64** | S | R | R | S | R | R | R | R | S | S | 18 |
| **44** | R | R | R | S | R | R | S | R | S | S | 19 |
| **46, 48** | R | R | R | S | R | R | S | R | R | S | 20 |
| **49** | R | R | R | S | R | R | S | S | R | S | 21 |
| **54** | R | S | R | S | R | R | R | S | R | S | 22 |
| **56, 57** | R | R | S | S | R | R | S | S | S | S | 23 |
| **61** | S | R | R | S | R | R | S | R | S | S | 24 |
| **1*** | R | S | R | R | R | R | S | R | R | S | 25 |
| **2*** | R | R | S | R | S | R | R | S | R | S | 26 |
| **3*** | R | S | S | S | S | R | R | S | S | S | 27 |
| **4*, 25*** | S | S | S | R | S | R | S | S | S | S | 28 |
| **5*** | R | S | S | R | S | R | S | S | R | S | 29 |
| **27*, 50*, 58*, 113*, 133*, 134*, 45#** | R | R | S | R | S | R | S | S | S | S | 30 |
| **43*, 60*** | R | S | R | R | S | R | S | S | S | S | 31 |
| **7*** | S | R | S | R | S | R | S | S | S | S | 32 |
| **10*, 11*, 22*** | R | S | S | S | S | R | S | R | S | S | 33 |
| **15*** | S | R | S | R | S | R | S | R | S | S | 34 |
| **16*, 17*, 163*, 27#** | R | R | R | R | S | R | S | R | S | S | 35 |
| **19*, 87#** | R | S | S | R | S | R | S | R | R | S | 36 |
| **21*** | R | R | R | S | R | S | R | R | S | S | 37 |
| **22*** | R | R | R | S | S | S | S | S | S | S | 38 |
| **23*, 24*, 26*** | R | R | R | S | R | S | S | S | S | S | 39 |
| **28*** | R | R | S | S | R | R | R | S | S | S | 40 |
| **30*** | R | S | R | S | S | S | R | S | S | S | 41 |
| **33*** | R | R | R | S | S | S | R | S | R | S | 42 |
| **34*** | R | R | R | S | S | S | R | S | S | S | 43 |
| **45*** | S | R | R | S | S | S | S | S | S | S | 44 |
| **46*** | R | S | R | S | R | S | R | R | R | S | 45 |
| **48*** | R | R | R | S | S | S | R | R | S | S | 46 |
| **49*** | R | R | S | S | R | S | R | S | R | S | 47 |
| **50*** | R | R | R | R | R | R | R | S | R | S | 48 |
| **61*** | R | S | S | S | S | S | R | R | R | S | 49 |
| **62*** | R | R | R | R | R | S | S | S | S | S | 50 |
| **1#, 5#, 8#, 16#, 17#, 22#, 23#, 24#, 25#, 28#, 29#, 30#, 33#, 34#, 35#, 36#, 37#, 38#, 40#, 67#, 68#, 69#, 70#, 72#, 73#, 74#, 73#, 79#, 81#, 82#, 83#, 84#, 86#, 87#, 89#, 91#, 92#, 95#, 96#, 98#, 99#, 100#, 101#, 102#, 103#, 104#, 105#, 42#, 43#, 49#, 50#** | R | R | S | R | S | R | S | R | S | S | 51 |
| **3#, 4#, 6#, 85#, 48#, 26^#^** | R | R | S | R | S | R | S | R | R | S | 52 |
| **1#, 32#, 10#, 31#, 46#** | R | R | S | R | S | R | R | R | R | S | 53 |
| **9#,14#** | R | R | S | R | R | R | R | R | R | S | 54 |
| **11#** | R | R | S | R | R | S | R | R | S | S | 55 |
| **12#,18#,19#** | R | R | S | R | S | S | R | R | S | S | 56 |
| **13#** | R | R | S | R | R | R | R | R | S | S | 57 |
| **15#** | R | R | R | R | R | R | R | R | S | S | 58 |
| **20#** | R | R | S | S | S | R | R | R | R | S | 59 |
| **31#** | R | R | S | R | S | R | R | R | R | S | 60 |
| **39#, 41#** | R | R | S | R | R | R | S | S | S | S | 61 |
| **47#** | R | S | S | R | S | S | S | S | R | S | 62 |
| **54#, 55#, 57#, 62#, 63#, 64#** | R | R | R | R | S | R | R | R | R | S | 63 |
| **66#, 88#, 90#, 71#** | R | S | S | R | S | R | S | S | S | S | 64 |
| **78#, 80#, 93#, 97#, 107#, 94#** | R | R | S | R | S | R | S | S | R | S | 65 |
| **60#, 61#, 106#** | R | R | S | R | S | R | R | R | S | S | 66 |
| **Virulence frequency %** | 6 | 13 | 75 | 22 | 80 | 2.8 | 75 | 39 | 70 | 100 |  |

***isolates during 1995-96 ^#^Isolates during 1997-98; rest of the isolates collected during 1994-95; a = Isolates of ascosporic ascosporic origin ; R= resistant response, S= susceptible response;**

**Table S2. Reaction of isolates of *Blumeria graminis tritici* on differential lines carrying known gene(s) for powdery mildew resistance (*Pm* genes) during 2015-19.**

| **Isolate number** | ***Pm1a*** | ***Pm1c*** | ***Pm2*** | ***Pm3a*** | ***Pm3b*** | ***Pm3c*** | ***Pm3d*** | ***Pm3f*** | ***Pm4a*** | ***Pm5a*** | ***Pm6*** | ***Pm8*** | ***Pm10*** | ***Pm12*** | ***Pm17*** | ***Pm25*** | ***Pm2+Mld*** | ***Pm10+15*** | ***Pm1+2+9+12*** | ***Pm5+?*** | ***Check lehmi*** | ***Pathotypes*** |
| --- | --- | --- | --- | --- | --- | --- | --- | --- | --- | --- | --- | --- | --- | --- | --- | --- | --- | --- | --- | --- | --- | --- |
| **1, 2, 3** | R | R | R | S | R | S | S | S | R | S | S | S | S | R | S | R | R | S | R | R | 3 | 1 |
| **4** | R | R | R | R | R | S | S | S | R | S | S | R | S | R | S | R | R | S | R | R | 4 | 2 |
| **5** | R | R | R | S | S | S | S | S | R | R | S | R | S | R | S | R | R | S | R | R | 3 | 3 |
| **6** | R | R | R | R | R | S | S | R | R | S | S | R | S | S | S | R | R | S | R | R | 4 | 4 |
| **7** | S | R | R | S | R | S | S | R | R | S | R | S | S | S | S | R | R | S | R | R | 4 | 5 |
| **8** | R | R | R | S | R | S | S | S | R | R | S | R | S | R | S | R | R | S | R | R | 3 | 6 |
| **9,14** | R | R | R | S | R | S | S | S | R | S | S | S | S | S | S | R | R | S | R | R | 3 | 7 |
| **10** | R | R | R | S | R | S | S | S | R | S | S | S | S | S | S | R | R | S | R | R | 4 | 8 |
| **11, 12, 16** | R | R | R | S | R | S | S | S | R | S | S | S | S | R | S | R | R | S | R | S | 4 | 9 |
| **13** | R | R | R | S | R | S | S | S | R | S | R | S | S | S | S | R | R | S | R | R | 4 | 10 |
| **15** | R | R | R | R | R | S | S | S | R | S | S | S | S | S | R | R | R | S | R | S | 4 | 11 |
| **22, 28** | S | R | R | S | R | S | S | S | R | S | S | S | S | S | S | R | R | S | R | S | 4 | 12 |
| **23, 24** | S | R | R | R | R | S | S | S | R | S | S | S | S | S | S | R | R | S | R | S | 4 | 13 |
| **25, 30** | S | S | R | R | R | S | S | S | R | S | S | S | S | S | S | R | R | S | R | S | 4 | 14 |
| **26, 27** | S | R | R | R | R | S | S | S | R | S | S | R | S | S | S | R | R | S | R | S | 4 | 15 |
| **29, 32, 33, 37** | S | R | R | R | R | S | S | S | R | S | S | S | S | S | S | R | R | S | R | R | 4 | 16 |
| **31** | S | S | R | R | R | S | S | S | S | S | S | S | S | S | S | S | R | S | R | S | 4 | 17 |
| **34** | S | R | R | R | R | S | S | R | R | S | R | S | S | S | S | R | R | S | R | S | 4 | 18 |
| **35** | S | R | R | R | R | S | S | S | S | S | S | S | S | S | S | R | R | S | R | S | 4 | 19 |
| **36, 48** | S | R | R | S | R | S | S | S | R | S | S | S | S | S | S | S | R | S | R | S | 4 | 20 |
| **38, 39, 44** | S | R | R | R | R | S | S | S | R | S | S | S | S | S | S | R | R | S | R | S | 4 | 21 |
| **40** | S | R | R | R | R | S | S | R | R | S | R | S | S | S | S | R | R | S | R | S | 4 | 22 |
| **41, 42, 50** | S | R | R | R | R | S | S | S | R | S | S | S | S | S | S | S | R | S | R | S | 4 | 23 |
| **43, 47, 49** | S | R | R | R | R | S | S | S | R | S | S | S | S | S | S | R | R | S | R | S | 4 | 24 |
| **45, 46** | S | R | R | S | R | S | S | S | S | S | S | S | S | S | S | S | R | S | R | S | 4 | 25 |
| **51** | S | R | R | R | R | S | R | S | R | S | R | S | S | S | R | R | R | S | R | S | 4 | 26* |
| **52** | R | R | R | R | R | R | R | R | R | S | R | S | S | S | R | R | R | S | R | S | 3 | 27^*^ |
| **53** | S | R | R | R | R | S | S | S | R | R | R | S | R | S | S | R | R | S | R | S | 4 | 28^*^ |
| **54** | R | R | R | R | R | R | R | S | R | S | R | S | S | S | R | R | R | S | R | R | 4 | 29^*^ |
| **55** | **S** | **R** | **R** | **R** | **R** | **S** | **S** | **S** | **S** | **S** | **R** | **S** | **S** | **S** | **S** | **S** | **R** | **S** | **R** | **S** | **4** | **30^*^** |
| **56** | S | R | R | R | R | S | R | S | R | S | S | S | S | S | S | R | R | S | R | S | 4 | 31^*^ |
| **57** | R | R | R | R | R | S | R | R | R | S | R | S | S | S | R | R | R | S | R | S | 4 | 32^*^ |
| **58** | S | R | R | R | R | S | R | R | R | S | R | S | S | S | R | R | R | S | R | S | 4 | 33^*^ |
| **59** | R | R | R | R | R | S | R | S | S | S | R | S | S | S | R | R | R | S | R | S | 4 | 34^*^ |
| **60** | R | R | R | R | R | S | R | S | R | S | R | S | S | S | R | R | R | S | R | R | 4 | 35^*^ |
| **61** | R | R | R | R | R | S | R | S | R | S | S | S | S | S | R | R | R | S | R | S | 4 | 36^*^ |
| **62** | R | R | R | R | R | R | R | R | R | S | S | S | S | R | R | R | R | S | R | S | 4 | 37^*^ |
| **63** | S | R | R | R | R | S | R | S | R | S | R | S | S | S | R | R | R | S | R | S | 4 | 38^*^ |
| **64** | R | R | R | R | R | S | R | S | R | S | R | S | S | S | R | R | R | S | R | S | 4 | 39^*^ |
| **65** | S | R | R | R | R | S | R | S | R | S | R | S | S | S | R | R | R | S | R | S | 4 | 40^*^ |
| **66, 67** | S | R | R | R | R | S | R | R | R | S | R | S | S | S | R | R | R | S | R | R | 4 | 41^*^ |
| **68, 69** | R | R | R | R | R | S | R | S | S | S | R | S | S | S | R | R | R | S | R | S | 4 | 42^*^ |
| **70** | S | R | R | R | R | S | R | S | R | S | S | S | S | S | R | R | R | S | R | S | 4 | 43^*^ |
| **17** | S | R | R | S | R | S | S | S | R | S | S | S | S | S | S | S | R | S | R | S | 4 | 44^*^ |
| **18** | S | R | R | S | R | S | R | S | R | S | S | S | S | S | R | S | R | S | R | S | 3 | 45^*^ |
| **19** | R | R | R | S | R | S | R | S | R | S | S | S | S | S | S | R | R | S | R | S | 4 | 46^*^ |
| **20** | S | R | R | S | R | S | S | S | R | S | S | S | S | S | S | R | R | S | R | S | 3 | 47^*^ |
| **21** | S | R | R | R | R | S | S | S | R | S | S | S | S | S | S | R | R | S | R | S | 4 | 48^*^ |
| **Virulence frequency (%)** | 63 | 4 | 0 | 33 | 1 | 96 | 71 | 87 | 9 | 96 | 71 | 91 | 99 | 86 | 73 | 16 | 0 | 100 | 0 | 71 | 100 |  |

Table S3 Details of the isolates of *Blumeria graminis tritici* collected during 2015-19

| **District** | **Location** | **Number of isolates collected** | **Number of ascosporic isolates** | **Total** |
| --- | --- | --- | --- | --- |
| Lahaul and Spiti | Kukumseri | 13 | 6 | 40 |
|  | Dalang Maidan | 12 | 9 |  |
| Kangra | Palampur | 5 | - | 14 |
|  | Bir Biling | 1 | - |  |
|  | Rakkar | 1 | - |  |
|  | Bankhandi | 1 | - |  |
|  | Dehra | 1 | - |  |
|  | Dehrian | 1 | - |  |
|  | Malan | 3 | - |  |
|  | Parour | 1 | - |  |
| Bilaspur | Namohal | 1 | - | 3 |
|  | Bilaspur | 1 | - |  |
|  | Ghagas | 1 | - |  |
| Una | Una | 1 | - | 7 |
|  | Gagret | 1 | - |  |
|  | Akrot | 1 | - |  |
|  | Pandoga | 1 | - |  |
|  | Bhaira | 1 | - |  |
|  | Mubarikpur | 1 | - |  |
|  | Amb | 1 | - |  |
| Chamba | Sehu | 1 | - | 2 |
|  | Sarol | 1 | - |  |
| Shimla | Shimla | 1 | - | 1 |
| Hamirpur | Bhaleth | 1 | - | 3 |
|  | Kakru | 1 | - |  |
|  | Sujanpur | 1 | - |  |

Table S4 Details of the isolates of *Blumeria graminis tritici* collected during 1994-98

| **District** | **Location** | **Number of conidial isolates** | **Number of ascosporic isolates** | **Total** |
| --- | --- | --- | --- | --- |
| Lahaul and Spiti | Kukumseri | 23 | 35 | 113 |
|  | Dalang Maidan | 30 | 25 |  |
| Kangra | Palampur | 5 | - | 46 |
|  | Bir Biling | 4 | - |  |
|  | Rakkar | 8 | - |  |
|  | Bankhandi | 4 | - |  |
|  | Dehra | 7 | - |  |
|  | Dehrian | 5 | - |  |
|  | Malan | 4 | - |  |
|  | Simplan | 5 | - |  |
|  | Parour | 4 | - |  |
| Bilaspur | Namohal | 4 | - | 10 |
|  | Bilaspur | 3 | - |  |
|  | Ghagas | 3 | - |  |
| Una | Una | 3 | - | 22 |
|  | Gagret | 3 | - |  |
|  | Akrot | 2 | - |  |
|  | Pandoga | 2 | - |  |
|  | Bhaira | 4 | - |  |
|  | Mubarikpur | 4 | - |  |
|  | Amb | 4 | - |  |
| Chamba | Sehu | 2 | - | 4 |
|  | Sarol | 2 | - |  |
| Shimla | Shimla | 2 | - | 2 |
| Hamirpur | Bhaleth | 3 | - | 10 |
|  | Kakru | 4 | - |  |
|  | Sujanpur | 3 | - |  |
| Mandi | Mandi | 2 | - | 8 |
|  | Jogindernagar | 6 | - |  |
